# Enzyme Inhibitors as Multifaceted Tools in Medicine and Agriculture

**DOI:** 10.3390/molecules29184314

**Published:** 2024-09-11

**Authors:** Sonia Del Prete, Mario Pagano

**Affiliations:** 1 Institute of Biosciences and Bioresources (IBBR), National Research Council (CNR), Via Pietro Castellino 111, 80131 Naples, Italy; 2 Institute of Research on Terrestrial Ecosystems (IRET), National Research Council (CNR), Via Madonna del Piano 10, Sesto Fiorentino, 50019 Florence, Italy; mario.pagano@cnr.it

**Keywords:** enzymes, inhibitors, plant serin protease, alpha-glucosidase, carbonic anhydrases

## Abstract

Enzymes are molecules that play a crucial role in maintaining homeostasis and balance in all living organisms by catalyzing metabolic and cellular processes. If an enzyme’s mechanism of action is inhibited, the progression of certain diseases can be slowed or halted, making enzymes a key therapeutic target. Therefore, identifying or developing enzyme inhibitors is essential for treating significant diseases and ensuring plant defense against pathogens. This review aims to compile information on various types of enzyme inhibitors, particularly those that are well studied and beneficial in both human and plant contexts, by analyzing their mechanisms of action and the resulting benefits. Specifically, this review focuses on three different types of enzyme inhibitors that are most studied, recognized, and cited, each with distinct areas of action and potential benefits. For instance, serine enzyme inhibitors in plants help defend against pathogens, while the other two classes—alpha-glucosidase inhibitors and carbonic anhydrase inhibitors—have significant effects on human health. Furthermore, this review is also intended to assist other researchers by providing valuable insights into the biological effects of specific natural or synthetic inhibitors. Based on the current understanding of these enzyme inhibitors, which are among the most extensively studied in the scientific community, future research could explore their use in additional applications or the development of synthetic inhibitors derived from natural ones. Such inhibitors could aid in defending against pathogenic organisms, preventing the onset of diseases in humans, or even slowing the growth of certain pathogenic microorganisms. Notably, carbonic anhydrase inhibitors have shown promising results in potentially replacing antibiotics, thereby addressing the growing issue of antibiotic resistance.

## 1. Introduction

**Figure 1:**
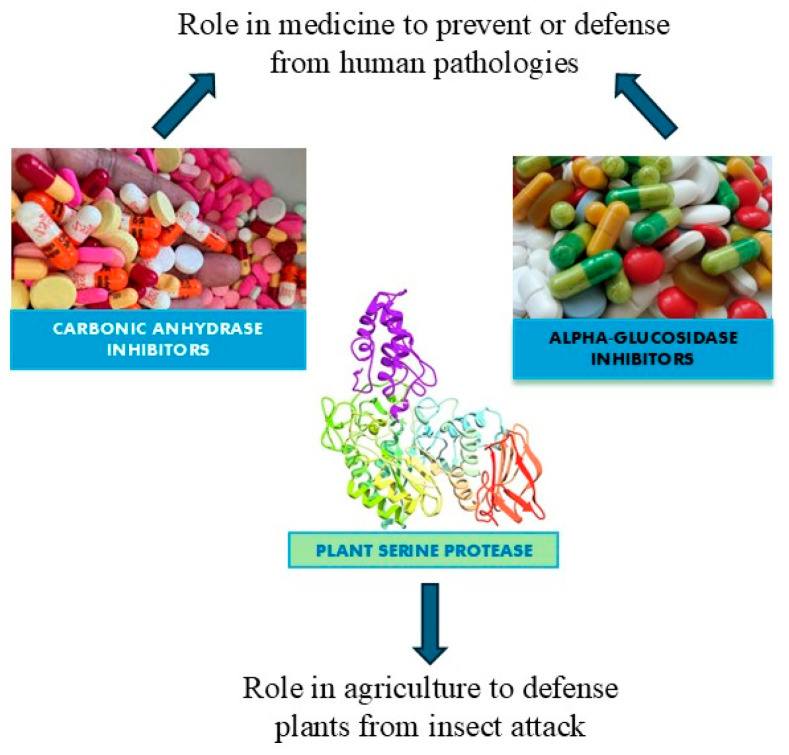
A diagram of the potential applications of the most studied enzyme inhibitors in medicine and agriculture.

Enzymes are biocatalysts that can speed up chemical reactions, but they are not changed by the reaction [1]. The two main types of enzyme inhibition are reversible inhibition and irreversible inhibition [2]. The interaction established between enzyme and inhibitor determines the difference. All the enzyme inhibitors act reversibly (weak, noncovalent interactions that are not permanent) or irreversibly (covalently alter the shape of the enzyme and/or active site) [3]. A reversible reaction is a chemical process in which the reactants form products that can subsequently react to regenerate the original reactants. Reversible reactions reach a state of equilibrium, where the concentrations of reactants and products remain constant over time. Additionally, reversible inhibition can be classified into competitive, non-competitive, and mixed inhibition depending on the location where the inhibitor binds to the enzyme [4]. Irreversible inhibition indicates an inhibitor capable of creating a chemical modification of the enzyme, usually by forming covalent bonds with the R groups of some amino acids. This interaction makes the enzyme inactive forever. Irreversible inhibition can occur anywhere in the enzyme. Instead, reversible inhibition involves an inhibitor capable of binding to the enzyme temporarily, either blocking its access or altering the shape of the active site. But if the inhibitor detaches from the enzyme, the effect can be reversed. In fact, when the inhibitor is detached, the enzyme returns to normal functioning [5]. Inhibitors can be beneficial or harmful to humans depending on the function of the enzymes being inhibited. Enzymes are essential for normal cellular functioning because they are the only way to slow down or turn off certain chemical reactions in the body. In addition, inhibitors can act as disease-curing drugs (if they inhibit essential enzymes used by foreign pathogens) or poisons (if they inhibit humans’ enzymes with vital functions) [6,7]. Enzyme inhibitors are found in nature, but their structures can be modified to produce them artificially in the laboratory. Naturally occurring enzyme inhibitors regulate numerous metabolic processes and are vital for life. Additionally, many naturally produced poisons are enzyme inhibitors that have evolved as toxic agents against predators, prey, and competing organisms. Artificial inhibitors, on the other hand, are commonly used as drugs and can also serve as insecticides, such as malathion, and herbicides. The primary use of enzyme inhibitors is in medicine, where they target human enzymes to correct pathological conditions. The use of natural inhibitors extracted from plants for treating type 2 diabetes and laboratory-designed inhibitors for addressing obesity and glaucoma represents a promising avenue for the future, potentially reducing the costs of current medications. Many pesticides are also enzyme inhibitors. Recent research on plant cystatins, such as Oryzacystatin I from rice and potato multicystatin and soybean CPI soyacystatin N, has shown their potential to inhibit pest growth. Moreover, soybean CPI soyacystatin N has also been demonstrated to suppress digestive enzyme activity. These findings suggest that plant cystatins could be effective and eco-friendly agents for pest control. In this review, three large classes of enzyme inhibitors have been analyzed which bring potential benefits to various living beings, along with their action. These enzyme inhibitors are among the most extensively studied and cited, particularly for their potential applications in agriculture and medicine. They can be categorized into the following groups: (a) plant serine protease inhibitors for their potential use in agriculture like plant defense, (b) alpha-glucosidase inhibitors from plants as potential candidates for the treatment of type 2 diabetes, and (c) carbonic anhydrase inhibitors as candidates for the treatment of glaucoma and obesity (Figure 1).

Many natural compounds have inhibitory effects on enzymes. For example, alkaloids, flavonoids, and terpenoids from plants exhibit significant enzyme inhibition properties, offering a potential source of new therapeutic agents. Plant Kunitz inhibitors, found abundantly in the *Fabaceae* (Leguminosae) family, are widely distributed in nature, encompassing the *Mimosoideae*, *Caesalpinioideae*, and *Faboideae*/Papilionoideae subfamilies [8,9]. These inhibitors serve as defensive proteins, exhibiting a range of activities outlined in this review, including antibacterial and antifungal properties, as well as impacting inflammation, coagulation, thrombosis, and cancer [9]. Studies on alpha-glucosidase inhibitors have shown that they can be used in a non-invasive treatment associated with mild and short-lasting effects and their side effects are gastrointestinal, including diarrhea, abdominal pain, and flatulence [10]. They represent a convenient method in the regulation of type 2 diabetes as they slow down the intestinal absorption of carbohydrates and simultaneously lower blood glucose levels [11]. Currently, only three alpha-glucosidase inhibitors are used: acarbose, miglitol, and voglibose. In recent decades, there has been a growing interest in the use of natural products and for this reason, there is a search for new inhibitors of natural origin to be used as therapeutic compounds, especially in the prevention and treatment of type 2 diabetes. To date, one of the approved drugs is metformin, an antidiabetic drug originally isolated from the herbal plant *Galega officinalis* [12]. The range of carbonic anhydrase inhibitors is very broad. They have various potential activities; in fact, they are effective in the treatment of bacterial, protozoan, and fungal infections such as helicobacter pylori, candida albicans, plasmodium falciparum, and mycobacterium tuberculosis [13,14,15]. In addition to this, carbonic anhydrase inhibitors may have pharmacological effects on the treatment of memory disorders and Alzheimer’s disease [16]. Blocking enzyme activity can impede the progression of diseases, making enzymes critical therapeutic targets. This review aims to explore three types of enzyme inhibitors, detailing their mechanisms of action and the benefits they could offer in biomedical and agricultural fields. It seeks to consolidate essential information on these inhibitors and to encourage further research into novel inhibitors, potentially leading to new developments and applications in diverse areas.

## 2. Protease Inhibitors in Plant Research

### 2.1. Plant Defenses

**Figure 2:**
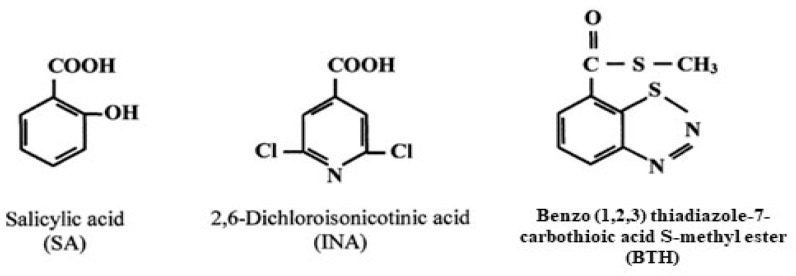
Formulas of commonly used chemical inducers of SAR.

When infected by pathogens, many plants develop increased resistance to subsequent attacks. This heightened resistance is known as systemic acquired resistance (SAR). In the SAR state, plants are primed to activate defense responses faster and more effectively upon subsequent pathogen encounters. Because SAR relies on the plant’s ability to utilize previous experiences, it serves as a model for the concept of “plant memory” in acquired disease resistance [17]. Many factors have been identified that can promote resistance in plants, including pathogens such as fungi, bacteria, and viruses that produce a hypersensitive necrotic reaction (HR). Moreover, non-virulent and weakened pathogenic strains, pests like insects and nematodes, biotic elicitors, and abiotic elicitors [e.g., chemical compounds such as benzothiadiazole (BTH), 2,6-dichloroisonicotinic acid (INA), salicylic acid, inorganic salts, etc., Figure 2] have also been reported to induce plant resistance [18,19,20,21,22,23]. Another possibility in plant defense strategy comes from protein inhibitors. The role of plant protease inhibitors as defense molecules against insect predation was highlighted for the first time by Green and Ryan in 1972. However, several trials have demonstrated the significant potential of these inhibitors to fight insect proliferation [24,25,26,27,28]. The primary defense mechanism of these inhibitors is their ability to suppress insect digestive enzymes, leading to insect resistance [17].

### 2.2. Definition and Classification of Protease Inhibitors 

Protease inhibitors are molecules that inhibit the function of proteases, which are enzymes that break down proteins by cleaving peptide bonds. By blocking the activity of proteases, these inhibitors are crucial in various biological processes. Protease inhibitors (PIs) are highly regarded as prime candidates with extensive applications in biotechnology and medicine. They play a crucial role in enhancing our understanding of fundamental protein interactions and in the development of novel compounds to manage pathological processes and various diseases [29,30]. The classification system introduced by Laskowski and Kato for PIs based on specific reactive sites established in their sequences can be categorized into four primary families: cysteine protease inhibitors; metalloprotease inhibitors; aspartic protease inhibitors; and serine protease inhibitors. A summary of the effects of these inhibitors is shown in Table 1. Regarding cysteine protease inhibitors, the literature refers also to phytocystatins which are the inhibitors of papain-like cysteine proteases. In plants, these inhibitors play a role in regulating developmental processes and responding to both abiotic and biotic stresses. Many plant pests utilize cysteine proteases to break down plant proteins, so cystatins can limit the resources available to these invaders. Consequently, research has primarily focused on the application of phytocystatins for pest control, with much less attention given to their specific internal functions within plants [26]. Metalloproteinase inhibitors work by chelating the metal ions present in the enzyme’s active site. An example could be actinonin and matlystatins which are highly effective metalloproteinase inhibitors, characterized by their unique N-hydroxy-2-pentyl-succinamic acid moieties [31]. Aspartic protease inhibitors (APIs) are proteins that block the catalytic function of aspartyl proteases, a type of protease characterized by an aspartate residue (Asp) in their active site [32]. APIs are essential for the normal physiological processes and life cycles of both eukaryotic and prokaryotic organisms. Furthermore, it is well known that aspartic proteases can facilitate the early stages of invasion by infectious organisms [32,33,34]. Regarding serine protease, among the significant diseases involving certain of these proteases, allergies—such as asthma—carry substantial clinical and economic burdens. β-Tryptase is a serine protease that plays a role in the pathogenesis of asthma and other allergic and inflammatory conditions, which are resistant to all known endogenous protease inhibitors. Consequently, there is considerable interest in developing highly potent and selective tryptase inhibitors, including those derived from plants [35]. For example, among the various plant species valued for their tryptase inhibitors, common flax (*Linum usitatissimum*) holds a prominent position. Flax seeds are well known for their ability to alleviate digestive disorders, stabilize blood sugar levels, enhance skin health, and inhibit the development of certain cancers [35].

### 2.3. Plant Protease Inhibitors

In plants, PIs play crucial roles in various biological processes [36,37,38]. They are involved in the utilization of storage proteins, the regulation of endogenous enzymatic activities, the variation of apoptosis and programmed cell death, and the maintenance of defense proteins or compounds against animals, insects, and microorganisms [38,39,40,41,42,43,44,45,46]. Laskowski and Kato [47] introduced a classification system for PIs based on specific reactive sites established in the sequences. However, in plants, PIs are further classified by their structural and biochemical properties into groups such as Bowman–Birk serine protease inhibitors, cereal trypsin/α-amylase inhibitors, cysteine protease inhibitors, metallocarboxypeptidase inhibitors, mustard trypsin inhibitors, potato type I inhibitors, potato type II protease inhibitors, serpins, soybean trypsin (Kunitz) inhibitors, and squash inhibitors [38,48,49]. A summary of the effects of these inhibitors is shown in Table 2. Rawlings et al. [50] proposed an updated classification system for PIs, organizing them into families and clans. This approach parallels the classification proposed by Laskowski and Kato [47] but aims to illustrate the evolutionary interactions among PIs. Proteins within the same clan share similar tertiary structures, and clans are further divided into families. Family members are grouped based on their origin, indicated by homologous amino acid sequences [38,51]. To establish the family to which a PI belongs, it is necessary to analyze the protein sequence within the inhibitory region. This area, known as the “inhibitory unit”, is part of the PI domain that interacts with the protease domain. Sometimes, the inhibitory unit may also encompass the reactive site (P1) of the PI. Usually, PIs from the same family inhibit a specific catalytic type of protease by a similar mechanism. However, certain families contain PIs that exhibit varying affinities towards different proteases or different types of proteases [38].

### 2.4. Examples of Applications of Plant Protease Inhibitors

Oryzacystatin I, a rice cystatin, inhibited the growth of the red flour beetle (Tribolium castaneum) [28,60], while potato multicystatin reduced the growth of western corn rootworm larvae [28,61]. Another example is soybean CPI soyacystatin N (scN) that suppressed the digestive enzyme activity and growth of the western corn rootworm and Colorado potato beetle [25,28,62]. These findings suggest that plant cystatins could be effective for eco-friendly pest control. However, the effectiveness of protease inhibitors in plant protection is complicated by the ability of insects to adapt to plant defenses. For example, protease inhibitors induced in potatoes and other plants through hormone application or transgene expression have often been ineffective [28,56,63,64]. The impact of dietary protease inhibitors on insects varies with their developmental stage, possibly due to the developmental regulation of multiple protease isoforms that vary in sensitivity to specific cystatins [28,61]. Insects can also produce multiple protease isoforms at a single developmental stage [25,28,65].

## 3. Alpha-Glucosidase Inhibitors from Plants as Potential Candidates for the Treatment of Type 2 Diabetes 

### 3.1. Diabetes Mellitus Type 2: Causes and Symptoms

Due to aging, urbanization, and poor eating habits leading to rampant obesity resulting in decreased physical activity, the number of people with diabetes and pre-diabetes in the world has increased exponentially [66]. Diabetes can be classified into two broad categories: type 1 and type 2 diabetes. Type 1 diabetes, also known as insulin-dependent diabetes, is one of the most common metabolic disorders occurring in childhood [67] In type 1 diabetes, there is a destruction of pancreatic beta cells mediated by T cells, inhibiting insulin production, and resulting in the onset of the disease [68]. On the other hand, type 2 diabetes is a condition that occurs due to a problem in the way the body regulates and uses sugar as fuel. This long-term condition causes an increase in circulating sugar in the blood, which in the long run can lead to disorders of the circulatory, nervous, and immune systems. In type 2 diabetes, the pancreas does not produce enough insulin, a hormone that regulates the movement of sugar, and the cells respond poorly to insulin and absorb less sugar. Type 2 diabetes used to be known as adult-onset diabetes, but both type 1 and type 2 diabetes can begin during childhood and adulthood. Type 2 is more common in older adults. But the increase in the number of obese children has led to more cases of type 2 diabetes among younger people. There is no cure for type 2 diabetes. Losing weight, eating well, and exercising can help manage the disease. If diet and exercise are not enough to control blood sugar, diabetes medications or insulin therapy may be recommended. Examples of common antidiabetic drugs that promote the above-mentioned effects include alpha-glucosidase inhibitors, metformin, and sodium–glucose co-transporter-2 (SGLT-2) inhibitors [69].

### 3.2. Alpha-Glucosidase: Structures and Biological Roles

**Figure 3:**
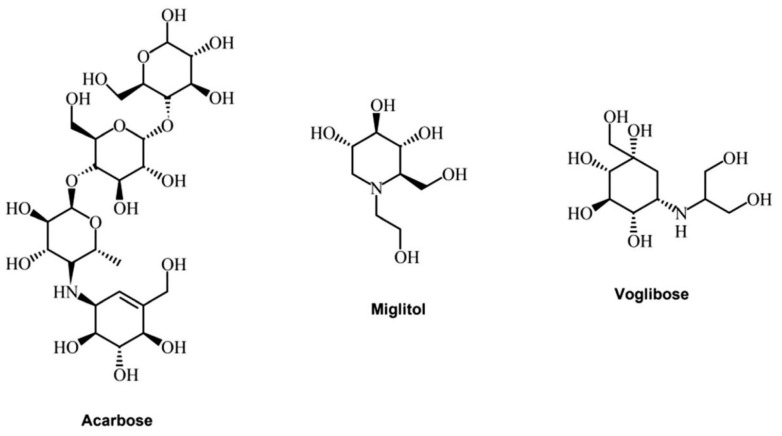
Acarbose, miglitol, and voglibose structures (elaborated from Phytochem Rev (2022) 21:1049–1079 [70]).

Alpha-glucosidase inhibitors (AGIs) reduce blood glucose levels and may thus prevent or delay type 2 diabetes mellitus (T2DM) and its associated complications in people at risk of developing T2DM. Currently, only three alpha-glucosidase inhibitors are utilized in clinical practice: acarbose, miglitol, and voglibose (Figure 3).

**Figure 4:**
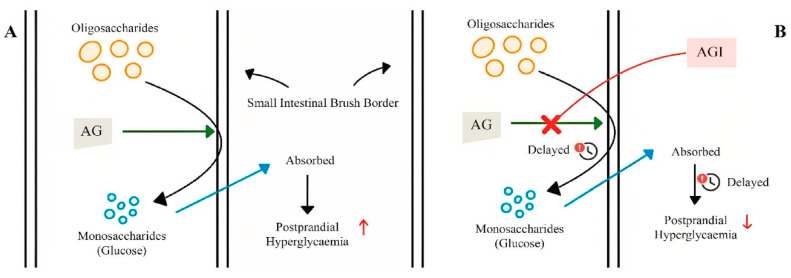
Mechanism of action of α-glucosidase inhibitors: (**A**) absence of α-glucosidase inhibitor; (**B**) presence of α-glucosidase inhibitor; AG: α-glucosidase; AGI: α-glucosidase inhibitor (from Mohammad Amil Zulhilmi Benjamin et al.; 2024) [74].

The many degradation reactions that occur in the gastrointestinal tract allow complex carbohydrates to be digested into monosaccharides, which are then absorbed in the small intestine. This process begins with the secretion of amylases (EC 3.2.1.1) produced mainly by the pancreatic and salivary glands leading to the hydrolysis of starch into shorter polysaccharides [71]. Upon entry into the small intestine, the partially hydrolyzed starch is further converted by pancreatic amylases that target the α-1,4 bonds of dextrins that release carbohydrates [72]. The final phase of carbohydrate metabolism is mediated by AGI (EC 3.2.1.20) in the brush border of enterocytes. The enzymes contain duplicated glycoside hydrolase domains (GH31) and catalyze the hydrolysis of the α-glucosidic bonds of disaccharides and oligosaccharides [73] (Figure 4).

AGI has been shown to be 1.5-fold overexpressed in non-insulin-dependent diabetic patients, contributing to increased postprandial glucose levels [75]. The action of a-amylase and AGI on polysaccharides and monosaccharides allows absorption at different speeds by the body; in particular, monosaccharide units are absorbed more rapidly. The inhibition of a-amylase and a-glucosidase activity may, therefore, delay the release of glucose from complex carbohydrates by modulating the onset of postprandial hyperglycemia, thus making it an ideal target for the management of type 2 diabetes. a-Amylase inhibitors are mainly present in plants, and the most studied molecules are glycoproteins isolated from beans. The structural similarity of these drugs to carbohydrates favors their attachment to the binding site of the a-glucosidase enzyme. Various types exist for the detection of alpha-glucosidase inhibition by natural and synthetic compounds. The colorimetry-based quantitative method is among the most common and practical approaches used to verify the inhibitory role of different compounds against the a-glucosidase enzyme. This method is based on measuring the amount of p-nitrophenol (pNP) released when p-nitrophenyl-a-D-glucopyranoside (pNPG) is hydrolyzed by the enzyme alpha-glucosidase. Subsequently, the absorbance of the product formed is measured spectrophotometrically and is characterized by a yellow color at 400 nm. Furthermore, a-glucosidase can also be detected thanks to a colorimetric/fluorometric glucose oxidase assay which allows the detection and quantification of the glucose released in the reaction.

### 3.3. Alpha-Glucosidase Extracted from Plants as Inhibitors

In recent decades, natural products have become an increasingly popular source of hypoglycaemic agents due to the common side effects and excessive cost of many antidiabetic drugs [76], especially in preventing and treating T2DM. Throughout history, medicinal plants and traditional remedies have been used to treat all medical conditions, including diabetes [77]. Numerous studies are currently being conducted on the mechanism of action and efficacy of these medicinal plants in treating diabetes. Plant-derived compounds represent a natural source of bioactive compounds that can be used for the development of effective drugs against diabetes mellitus. M.A.Z. Benjamin et al. showed a summary of medicinal plant extracts showing in vitro α-glucosidase inhibition activity according to Southeast Asia. Numerous studies have been conducted to isolate various active constituents from distinct parts of the chosen medicinal plants. Interestingly, although the same species were isolated, they yielded different active constituents, leading to distinct results. This was evident in the case of *C. excavata*, where the isolation of carbazomarin-C and dentatin resulted in varying IC 50 values for α-glucosidase inhibition activity. Thant et al. isolated and identified different extracts of these plants, with seven among them being newly discovered compounds [78]. In total, 19 pure active ingredients have been isolated and successfully identified from different extracts of these plants, of which 7 are newly discovered compounds. Cucurbitaceae family member *Momordica charantia* L. has been exploited as a traditional medicine for managing diabetes mellitus and other metabolic syndromes. In fact, Hussain, F. described how *M. charantia* methanolic extract shows potent α-glucosidase inhibition activity and significantly improves fasting blood glucose levels and insulin in diabetic rats. The acarbose shows higher α-glucosidase inhibition (79.91 ± 0.77%) in vitro than *M. charantia* methanolic extract (72.30 ± 0.30%) [79]. Kim, H.R et al. demonstrated that *A. villosum* water extract was investigated for its α-glucosidase activity at different concentrations of 1, 3, and 5 mg/mL, which proportionally increased the inhibition against rat AGI with the IC50 of 31.99 ± 6.79%, 48.85 ± 4.75%, and 62.58 ± 6.69%, respectively. Although *A. villosum* water extract has lower inhibition on α-glucosidase than the reference acarbose, it showed a considerable drop in blood glucose levels in the sucrose loading test when administered to the rats compared to the control group [80]. Moreover, Barber et al. described the potential of selected flavonoids to inhibit human intestinal α-glucosidases, hence slowing carbohydrate digestion and reducing postprandial glycemia. A sensitive and accurate method to determine sugar hydrolysis by sucrase, maltase, and isomaltase has been successfully developed and validated. The role and the use of HPAE-PAD to detect subtle changes in the concentrations of five sugars simultaneously, with minimal sample preparation and high precision within 32 min, is very important [81]. Acarbose and flavonoids exhibit different inhibition of human enzymes to those reported for yeast or mammalian α-glucosidases, emphasizing the need for a more pragmatic screening approach on individual human enzymes to elucidate their actual inhibitory potentials in vivo. Flavonoids from various sources are more effective against α-glucosidase than α-amylase. The low solubility of some flavonoids limits the experimental concentration which can be employed, preventing the determination of IC50 values and necessitating the use of IC25 or IC15 values instead [81].

## 4. Carbonic Anhydrase Inhibitors as Candidates for the Treatment of Glaucoma and Obesity

### 4.1. Mechanisms of Action of Carbonic Anhydrases 

In our world, where life is based on carbon, carbon dioxide transforming through a hydration reaction is a fundamental transformation. Carbon dioxide is converted to bicarbonate by a reaction chemically represented in the following equation: CO_2_ + H_2_O ⇆ HCO_3_ + H^+^. In nature, this reaction is very slow. Carbonic anhydrase enzymes (CA; EC 4.2.1.1) are placed in this context, allowing the acceleration of this reaction to meet biological needs. The characteristics of exclusivity and selectivity applied to biological targets fit perfectly with the recipe for the success of medicinal chemistry, and ACs are a typical example of this. It is recognized that CAs can be used for the treatment of various diseases, also showing interesting results in the field of anti-infectives and central nervous system (CNS) diseases, and are also usable in the biotechnological field [82].

### 4.2. Carbonic Anhydrase: Their Isoforms and Inhibitors 

Carbonic anhydrase (EC 4.2.1.1) is an enzyme present in all living organisms that catalyzes a reversible reaction of hydration of CO_2_ with the formation of bicarbonate [82]. Carbonic anhydrase (CA) includes eight different families such as α-CA, β-CA, γ-CA, δ-CA, ζ CA, η-CA, θ CA, and ι-CA [83]. Primarily, only the α-CA isoforms are present in the cytoplasm of green plants, algae, vertebrates, and bacteria [84]. Human CAs have 16 different isoforms (hCA). hCA I and II represent the cytosolic forms, present in the red blood cells, osteoclasts, eye, gastrointestinal tract, lung, brain, and testes. Their presence inside the red blood cells allows the maintenance of a physiological pH of the human blood producing the bicarbonate ion [85]. Furthermore, the hCA II plays an important role in some metabolic pathways and is, therefore, implicated in diseases such as glaucoma, renal tubule acidosis, and osteoporosis. CA III is also a cytosol-bound isoform expressed in tissues such as skeletal muscle and white and brown adipose tissues. It is, therefore, directly related to adipogenesis due to its unique ability to act as a regulator of peroxisome proliferator-activated receptor g2 (PPARg2) expression [86]. CA IV is a membrane-bound isoform expressed in the lung, kidney, brain, and eye and is important in the progression of various diseases such as retinitis pigmentosa. CA VA and CA VB are the only isoforms expressed in mitochondrial hepatocytese adipocytes, respectively. CA VA is present in the liver and supplies bicarbonate ions to be used by carbamylphosphate synthetase I and this leads to ureagenesis [87]. Both enzymes can be considered valuable drug targets for the treatment of obesity and insulin resistance by modulating both gluconeogenesis and lipogenesis [88]. The CA VI isoform is the secreted CA isoform, present in tears, in the respiratory airways, in the epithelial lining of the alimentary canal, in enamel organs, and, more extensively, in human saliva. The CAVI isoform is required to maintain the homeostasis of the mouth and adequate pH level in the saliva [89]. The inhibition of the CA VI isoform causes the loss of taste or sometimes irregularity in the perception of taste. The CA VI isoform can be inhibited in diseases such as dental caries. The CA VII isoform is expressed in the liver, brain, skeletal muscles, and colon. It is a cytosolic isoform that exists in two forms: one form has the absolute amino acid sequence while the other contains an N-terminal truncation of 56 residues. Likewise, CA VII has two glutathionylated surface cysteine residues as CA III to protect against cellular oxidative damage. The inhibition of CA VII results in and is considered to suppress neuronal excitation as a valuable target for the treatment of seizures and neuropathic disorders pain. CA IX is a transmembrane isoform expressed in GI mucosa and tumors [90]. CA IX mediates several processes such as tumourigenesis, pH control, proliferation and migration of tumor cells, and cell adhesion. High CA IX expression is tumor-regulated hypoxia which in turn has established itself as a predictive factor for the growth of a variety of tumors [91]. The inhibition of CA IX represents a potential target of anticancer drugs. Similar to this, the CA XII isoform has also been implicated in the progression of multiple tumors, but unlike CA IX, it is not established as a predictive factor. CA XII is a transmembrane isoform expressed in various organs such as the eye, tumors, reproductive epithelia, prostate, ovaries, intestines, and kidney. Due to its expression in various organs, its role has been implicated in a wide range of cancers like breast cancer, prostate cancer, etc. It is important for normal kidney function and also found a role in glaucoma [92]. CA XIII is expressed in organs like the kidney, thymus, submandibular glands, small intestine, and reproductive organs. It is also an active cytosolic isoform of CA. It has been suggested that CA XIII has a crucial role in the pH regulation of reproductive processes including sperm mobility. CA XIV is a transmembrane isoform of CA. It is expressed in various organs like the brain, small intestine, urinary bladder, kidney, and colon and shows high sequence similarity with CA XII [93]. There are various types of inhibitors of pharmacological origin and synthetic origin. Carbonic anhydrase inhibitors reduce the activity of carbonic anhydrase. This reduces the resorption of bicarbonate from the proximal tubule in the kidneys, which causes a direct increase in bicarbonate excretion and mild increases in sodium, and potassium excretion. Generally, the electrolyte effects of carbonic anhydrase inhibitors are mild and they are typically not used for their diuretic capacity. Acetazolamide, dichlorphenamide, and methazolamide are carbonic anhydrase inhibitors. Carbonic anhydrase inhibitors also decrease the secretion of aqueous humor (the aqueous humor is the clear fluid that fills the space between the lens and the cornea of the eyeball), which results in a decrease in intraocular pressure [94]. Carbonic anhydrase inhibitors are mainly used for the treatment of glaucoma or other ocular conditions where the lowering of the intraocular blood pressure has been deemed beneficial. Acetazolamide (AZA) is also used for the treatment and prevention of acute mountain sickness (also known as altitude sickness) and in some types of epilepsy [95]. Dichlorphenamide may be used to treat certain inherited muscle disorders. Carbonic anhydrase inhibitors may be also used in the treatment of other conditions [96].

### 4.3. Inhibitors of Carbonic Anhydrases in the Treatment of Glaucoma

The causes of vision loss could include elevated intraocular pressure (IOP) that can damage the optic nerve. The treatment for glaucoma is to lower the pressure within the eye, called “target pressure”, to a level that is unlikely to cause further damage to the optic nerve [97]. Target blood pressure varies between individuals and may also change during the course of treatment. For the treatment of glaucoma, there are different types of drugs, including β-blockers and prostaglandin receptor antagonists [98]. The problem with the existing drugs includes failure to achieve target IOP, drug-related side effects, and poor patient compliance with short-acting molecules. Since the disease is on the increase, it is important to explore new drugs that could satisfy all the needs listed above and also be less expensive than drugs on the market. In recent years, new types of sulfonamide CAIs with good water solubility and IOP-lowering effects have been developed with the chemical “tail” approach, which consists of attaching suitable groups or scaffolds to aromatic or heteroaromatic sulfonamides. Typically, the inhibitors of this type are used alone or in combination with other drugs such as PG analogs or adrenergic agonists/antagonists. CAI drugs are very reliable in reducing intraocular pressure, leading to a decrease in the fluid that the eye continuously produces, called aqueous humor. There are two types of medications: topical medications include dorzolamide and brinzolamide, and oral medications include acetazolamide and methazolamide.

### 4.4. Inhibitors of Carbonic Anhydrases in the Treatment of Obesity

Obesity, currently considered a chronic and degenerative disease, is a multifactorial and widespread medical problem [99] which presents a large number of metabolic and psychological comorbidities making it difficult to manage, both from a medical and social perspective. Some of these comorbidities include metabolic dysfunctions (type 2 diabetes, fatty liver disease, dyslipidemia, gallstones, and gout); cardiovascular diseases (atherosclerosis, hypertension, atrial fibrillation, and heart failure); increased chance of getting cancer (including colon, breast, and pancreatic cancers); lung problems (sleep apnea and asthma); mental problems (cognitive impairment, depression, anxiety, and panic disorders), and many other inconveniences. The field of anti-obesity CAIs is still under constant exploration, so three main approaches can currently be envisaged, which have produced several interesting developments in the last two decades: (1) the repurposing of drugs originally discovered for pharmacological applications other than obesity; (2) search for natural products using virtual screening procedures or more classic enzyme inhibition assays; and (3) de novo drug design based on already identified leads or on the structural biology data of inhibitory enzyme adducts characterized in detail, mainly by X-ray crystallographic techniques [100]. The use of AZA, ZNS, and TPM as anti-obesity drugs represents an interesting and successful, yet also problematic, example of drug repurposing. This is especially true considering their use either alone or in combination with other agents such as phentermine, bupropion, and metformin [101]. Sari C. was demonstrated to induce weight loss in many obese patients, also improving their blood glucose levels [102]. How do these agents exert their anti-obesity beneficial effects? Although the pharmacology of TPM and ZNS is rather complex as these compounds bind to a multitude of targets, both of them, and obviously AAZ, are effective CAIs against the human CA (hCA) isoforms involved in fatty acid biosynthesis/DNL [103]. There are many classes of such potent and also selective mitochondrial CA inhibitors, apart from the sulphonamides and sulfamates reported earlier, which strengthen the rationale of using topiramate and zonisamide as anti-obesity agents, alone or in combination with other drugs, with all their limitations mentioned above due to the side effects correlated or not with off-target CA inhibition.

## 5. Conclusions

Enzyme inhibitors are essential for the health of all living organisms as they regulate enzyme activity, aiding in disease treatment and enhancing defense mechanisms. Understanding their mechanisms of action and therapeutic benefits is crucial for developing new and effective inhibitors. Enzyme inhibitors occur naturally and can also be synthesized in laboratories. Naturally occurring inhibitors regulate many essential metabolic processes and have evolved as poisons in some organisms, serving as toxic agents against predators, prey, and competitors. Artificial inhibitors, commonly used as drugs, also have applications as insecticides and herbicides. The most prevalent use of enzyme inhibitors is in pharmaceuticals, where they target human enzymes to correct pathological conditions. As discussed in this review, carbonic anhydrase inhibitors (CAIs) and alpha-glucosidase inhibitors (AGIs) are used therapeutically as anti-obesity and anti-glaucoma agents, respectively, as well as antidiabetic agents. This is also a significant area of focus in pharmaceutical research, allowing the development of alternative molecular targets to antibiotics and partially mitigating the issue of antibiotic resistance. On the other hand, we have highlighted the application of plant serine protease inhibitors in agriculture. The use of eco-friendly, sustainable, and effective protein molecules to disrupt or slow down nutrient metabolism in pests is a practical approach to insect pest management in crops. Like the other two types of inhibitors described here, plant serine protease inhibitors also warrant further exploration regarding their mode of action, biological implications, and applications. In fact, plant inhibitors could be a promising approach to overcoming the development of resistance. In conclusion, this review compiles and synthesizes information on various enzyme inhibitors, emphasizing their potential to revolutionize the future of drug development in human and plant biomedical fields. It serves as a valuable guide for researchers studying the biological effects of natural or synthetic inhibitors.

## Figures and Tables

**Table 1:** The table summarizes the key protease inhibitors mentioned and their effects.

Protease Inhibitor	Type	Effect	Bibliography
Phytocystatins	Cysteine protease inhibitor	Inhibits papain-like cysteine proteases, regulates developmental processes, and responds to stresses.	U.Schlüter (https://doi.org/10.1016/j.sajb.2009.02.101) [26]
Actinonin	Metalloprotease inhibitor	Chelates metal ions in the enzyme’s active site.	Leipoldt et al. (https://doi.org/10.1038/s41467-017-01975-6) [31]
Matlystatins	Metalloprotease inhibitor	Chelates metal ions in the enzyme’s active site.	Leipoldt et al. (https://doi.org/10.1038/s41467-017-01975-6) [31]
Aspartic protease inhibitors (APIs)	Aspartic protease inhibitor	Blocks the catalytic function of aspartyl proteases, essential for physiological processes and life cycles.	Osmani et al., 2022; Stewart K et al., 1999; Cater et al., 2002 [32,33,34]
Tryptase inhibitors	Serine protease inhibitor	Flax (Linum usitatissimum) is noted for tryptase inhibitors and various health benefits.	Rosu et al., 2010 [35]

**Table 2:** The table summarizes the protease inhibitors mentioned in the text and their effects.

Type of Inhibitor	Effect	Bibliography
Bowman–Birk Serine Protease Inhibitors	Suppress inflammation and protease activity; show potential in cancer chemoprevention and multiple sclerosis treatment.	https://doi.org/10.3390/ph13120421 [52]
Cereal Trypsin/α-Amylase Inhibitors	Suppress the activities of enzymes that degrade nutrients: α-amylase (which breaks down starch) and trypsin (which breaks down proteins), and are among the various groups of wheat seed proteins that contribute to natural pest and pathogen defense.	https://doi.org/10.1007/s00394-022-02841-y [53]
Cysteine Protease Inhibitors	Cysteine proteases have been identified as crucial enzymes in the regulation of programmed cell death in animals.	https://doi.org/10.1105/tpc.11.3.431 [54]
Metallocarboxypeptidase Inhibitors	Metallocarboxypeptidase inhibitors are produced in response to stress and play a role in defending against pests.	https://doi.org/10.3390/molecules25030700 [55]
Mustard Trypsin Inhibitors	They contribute to plant protection.	https://doi.org/10.1016/s0965-1748(00)00164-8 [56]
Potato Type I Protease Inhibitors	Modulating plant physiology (e.g., dehydration response, programmed cell death, plant growth, trichome density, and branching) and host resistance.	https://doi.org/10.2174/138920311796391151 [57]
Potato Type II Protease Inhibitors	Modulating plant physiology (e.g., dehydration response, programmed cell death, plant growth, trichome density, and branching) and host resistance.	https://doi.org/10.2174/138920311796391151 [57]
Serpins	They can be responsible for cell death and pathogen-associated stress.	https://doi.org/10.3390/ijms20061345 [38]
Soybean Trypsin (Kunitz) Inhibitors	They are known to play a protective role against herbivores.	https://doi.org/10.3389/fpls.2023.1129454 [58]
Squash Inhibitors	Potent canonical serine proteinase inhibitors isolated from Cucurbitaceae	https://doi.org/10.2174/1389203043379477 [59]

## Data Availability

Not applicable.

## Abbreviations

AGI	(alpha-glucosidase)
API	(aspartic protease inhibitors)
BABA	(beta amminobutyric acid)
BTH	(benzothiazole)
CA	(carbonic anhydrase)
GH 31	(glycoside hydrolase domains)
HR	(hypersensitive reaction)
INA	(2,6 dichlorosomicotomic acid)
IOP	(intraocular pressure)
PI	(protease inhibitor)
SAR	(systemic acquired resistance)
SGLT-2	(sodium–glucose 6-transporter)
T2DM	(type 2 diabetes mellitus)
